# New Anti-Inflammatory Cembranes from the Cultured Soft Coral *Nephthea columnaris*

**DOI:** 10.3390/md13063443

**Published:** 2015-05-29

**Authors:** Ting-Hsi Hsiao, Chun-Sung Sung, Yu-Hsuan Lan, Yi-Chen Wang, Mei-Chin Lu, Zhi-Hong Wen, Yang-Chang Wu, Ping-Jyun Sung

**Affiliations:** 1 Graduate Institute of Marine Biology, National Dong Hwa University, Pingtung 944, Taiwan; E-Mails: hsiaoinon@gmail.com (T.-H.H.); jinx6609@nmmba.gov.tw (M.-C.L.); 2 National Museum of Marine Biology and Aquarium, Pingtung 944, Taiwan; 3 Department of Anesthesiology, Taipei Veterans General Hospital, Taipei 112, Taiwan; E-Mail: sung6119@gmail.com; 4 School of Medicine, National Yang-Ming University, Taipei 112, Taiwan; 5 School of Pharmacy, College of Pharmacy, China Medical University, Taichung 404, Taiwan; E-Mail: lanyh@mail.cmu.edu.tw; 6 Department of Marine Biotechnology and Resources, Asia-Pacific Ocean Research Center, National Sun Yat-sen University, Kaohsiung 804, Taiwan; E-Mails: cvyc.wang@gmail.com (Y.-C.W.); wzh@mail.nsysu.edu.tw (Z.-H.W.); 7 Division of Cardiology, Department of Internal Medicine, Kaohsiung Armed Forces General Hospital, Kaohsiung 802, Taiwan; 8 Chinese Medicine Research and Development Center, China Medical University Hospital, Taichung 404, Taiwan; 9 Graduate Institute of Natural Products, Kaohsiung Medical University, Kaohsiung 807, Taiwan; 10 Center for Molecular Medicine, China Medical University Hospital, Taichung 404, Taiwan

**Keywords:** *Nephthea columnaris*, cembrane, octocoral, antiinflammatory, iNOS, COX-2, cytotoxicity

## Abstract

Two new cembranes, columnariols A (**1**) and B (**2**), were isolated from the cultured soft coral *Nephthea columnaris*. The structures of cembranes **1** and **2** were elucidated by spectroscopic methods. In the anti-inflammatory effects test, cembranes **1** and **2** were found to significantly inhibit the accumulation of the pro-inflammatory iNOS and COX-2 protein of the lipopolysaccharide (LPS)-stimulated RAW264.7 macrophage cells. Compound **1** exhibited moderate cytotoxicity toward LNCaP cells with an IC_50_ value of 9.80 μg/mL.

## 1. Introduction

**Scheme 1:**
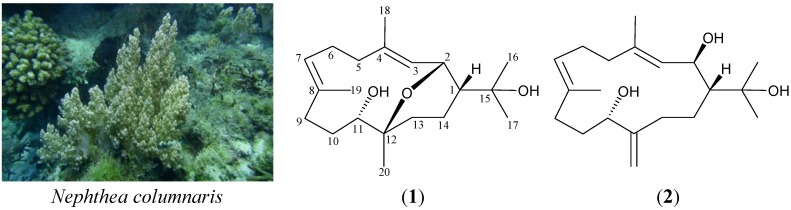
The soft coral *Nephthea columnaris* and the structures of columnariols A (**1**) and B (**2**).

Among the natural products isolated from soft corals, the cembranes are major representative compounds. Previous investi*g*ations of soft corals belonging to the genus *Nephthea* (family Nephtheidae), collected off the waters of Taiwan, have yielded several interesting cembranes [1,2]. In our continuing studies, a sample originally collected off the coast of Southern Taiwan, identified as *Nephthea columnaris* (Studer, 1895), yielded two new cembranes, columnariols A (**1**) and B (**2**) (Scheme 1). In this paper, we deal with the isolation, structure determination and bioactivity of cembranes **1** and **2**.

## 2. Results and Discussion

Columnariol A (**1**) was isolated as a colorless oil and the molecular formula for this compound was determined to be C_20_H_34_O_3_ (four unsaturations) using HRESIMS (C_20_H_34_O_3_ + Na, *m*/*z* 345.23989, calcd 345.24002). Comparison of the ^13^C-NMR and DEPT data with the molecular formula indicated there must be two exchangeable protons, which required the presence of two hydroxyl groups. This deduction was supported by a broad absorption in the IR spectrum at 3419 cm^−1^. The ^13^C-NMR data for **1** confirmed the presence of 20 carbon signals (Table 1), characterized by DEPT spectrum as five methyls, six sp^3^ methylenes, three sp^3^ methines (including two oxymethines), two oxygenated sp^3^ quaternary carbons, two sp^2^ methines and two sp^2^ quaternary carbons. Based on the ^1^H and ^13^C-NMR spectra (Table 1), **1** was determined to contain two isolated methyl-bearing trisubstituted double bonds (δ_H_ 5.48, 1H, d, *J* = 10.8 Hz, H-3; 5.02, 1 H, dd, *J* = 10.8, 4.4 Hz, H-7; 1.69, 3 H, s, H_3_-18; 1.60, 3 H, s, H_3_-19; δ_C_ 126.4, CH-3; 139.1, C-4; 123.8, CH-7; 136.2, C-8; 15.3, CH_3_-18; 17.3, CH_3_-19). Thus, from the reported data, the proposed skeleton of **1** was suggested to be a cembrane with two rings.

**Table 1:** ^1^H (400 MHz, CDCl_3_) and ^13^C (100 MHz, CDCl_3_) NMR data, ^1^H–^1^H COSY and HMBC correlations for cembrane **1**.

Position	δ_H_ (*J* in Hz)	δ_C_, Multiple	^1^H–^1^H COSY	HMBC
1	1.79 m	49.9, CH	H-2, H_2_-14	C-2, -3
2	4.65 dd (10.8, 4.4)	70.1, CH	H-1, H-3	C-1, -3, -4, -12, -14
3	5.48 d (10.8)	126.4, CH	H-2, H_3_-18	C-2, -5, -18
4		139.1, C		
5	2.20 ddd (12.4, 3.6, 3.6);1.98 m	40.0, CH_2_	H_2_-6	C-3, -4, -6, -7, -18
6	2.31 ddd (14.0, 9.6, 3.6); 2.15 m	25.1, CH_2_	H_2_-5, H-7	C-4, -7, -8
7	5.02 dd (10.8, 4.4)	123.8, CH	H_2_-6, H_3_-19	C-9, -19
8		136.2, C		
9	2.16 m; 1.99 m	35.3, CH_2_	H_2_-10	C-7, -8, -10, -11, -19
10	1.82 m; 1.29 m	31.6, CH_2_	H_2_-9, H-11	C-8, -9, -11, -12
11	3.84 d (9.2)	72.4, CH	H_2_-10	C-9, -12, -20
12		75.3, C		
13	2.46 ddd (14.0, 3.6, 3.6); 1.39 m	33.7, CH_2_	H_2_-14	C-1, -11, -12, -14, -20
14	1.75 m; 1.66 m	16.0, CH_2_	H-1, H_2_-13	C-1, -2, -12, -13
15		71.5, C		
16	1.17 s	28.3, CH_3_		C-1, -15, -17
17	1.12 s	27.8, CH_3_		C-1, -15, -16
18	1.69 s	15.3, CH_3_	H-3	C-3, -4, -5
19	1.60 s	17.3, CH_3_	H-7	C-7, -8, -9
20	1.04 s	24.0, CH_3_		C-11, -12, -13

From the ^1^H–^1^H COSY spectrum of **1** (Table 1), it was possible to differentiate among the separate spin systems of H-3/H-2/H-1/H_2_-14/H_2_-13, H_2_-5/H_2_-6/H-7 and H_2_-9/H_2_-10/H-11. These data, together with the HMBC correlations between H-1/C-2, -3; H-2/C-1, -3, -4, -14; H-3/C-2, -5; H_2_-5/C-3, -4, -6, -7; H_2_-6/C-4, -7, -8; H-7/C-9; H_2_-9/C-7, -8, -10, -11; H_2_-10/C-8, -9, -11, -12; H-11/C-9, -12; H_2_-13/C-1, -11, -12, -14; and H_2_-14/C-1, -2, -12, -13, observed in an HMBC experiment, established the connectivity from C-1 to C-14 in a 14-membered ring. The vinyl methyls attached at C-4 and C-8 were confirmed by the HMBC correlations between H-3, H_2_-5/C-18; H_3_-18/C-3, -4, -5; and H-7, H_2_-9/C-19; H_3_-19/C-7, -8, -9, and were further supported by the allylic couplings between H-3/H_3_-18 and H-7/H_3_-19. An isopropyl carbinol group at C-1 was elucidated by the HMBC correlations between H_3_-16/C-1, -15, -17 and H_3_-17/C-1, -15, -16. The HMBC correlations between H-2 (δ_H_ 4.65) and C-12, an oxygenated quaternary carbon at δ_C_ 75.3; and H_3_-20/C-11, -12, -13, suggested an oxygen atom had to be positioned between C-2 and C-12 to form an ether bridge. Thus, the remaining hydroxyl group was positioned at C-11, an oxygen-bearing methine.

**Figure 1:**
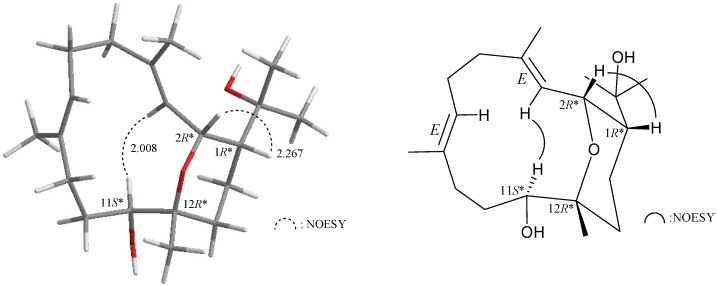
Key NOESY correlations of compound **1**.

As those of the cembranoid derivatives isolated from Formosan soft coral [3], the relative configuration of compound **1** was deduced from NOESY correlations and Chem3D Ultra 9.0 (Figure 1). If H-1 assumes a β-orientation and this proton correlated with H-2, but not with H-3, and a large coupling constant was recorded between H-2 and H-3 (*J* = 10.8 Hz), indicated that the dihedral angle between H-2 and H-3 is approximately 180° and the configurations of both chiral carbons C-1 and C-2 were assigned as R*. Furthermore, H-11 correlated with H-3, but not with H_3_-20, indicating that the chiral carbons C-11 and C-12 can be assigned as S* and R*, respectively. No correlation was found between H-3/H_3_-18 and H-7/H_3_-19, indicating that C-3/4 and C-7/8 carbon-carbon double bonds had an *E*-configuration, by modeling analysis.

Cembrane **2** (columnariol B), obtained as a colorless oil, showed an [M + Na]^+^ signal at *m*/*z* 345.23977 in the HRESIMS, suggesting the molecular formula C_20_H_34_O_3_ (calcd C_20_H_34_O_3_ + Na, 345.24002), with four units of unsaturation. The IR absorption of **2** at 3355 cm^−1^ indicated the presence of hydroxyl functionality. ^1^H and ^13^C-NMR spectral data (Table 2) showed that the structure of **2** contained an isopropyl carbinol, two isolated methyl-bearing trisubstituted olefins, an exocyclic olefin, an aliphatic methine carbon, two oxymethine carbons and six methylene carbons. These data suggested that **2** possessed a cembrane skeleton with functionalities of an isopropyl carbinol, two isolated methyl-bearing trisubstituted double bonds, an exocyclic double bond and two hydroxy groups. ^1^H–^1^H couplings in the COSY spectrum of **2** enabled identification of the C-3/-2/-1/-14/-13, C-5/-6/-7, C-9/-10/-11, C-3/-18 (by allylic coupling) and C-11/C-20 (by allylic coupling) units (Table 2), which were assembled with the assistance of an HMBC experiment. In the HMBC experiment of **2**, the isopropyl carbinol group at C-1 was confirmed by correlations between H_3_-16/C-1, -15, -17 and H_3_-17/C-1, -15, -16. The vinyl methyl groups attached at C-4 and C-8 were confirmed by correlations between H-3/C-18; H_3_-18/C-3, -4, -5; and H-7, H_2_-9/C-19, H_3_-19/C-7, -8, -9, respectively. An exocyclic carbon-carbon double bond at C-12 was confirmed by the correlations between H_2_-13/C-20 and H_2_-20/C-11, -12, -13. Thus, the remaining hydroxyl groups had to be positioned at C-2 and C-11, the oxymethines at δ_C_ 71.7 and 70.8. Based on the above findings, the planar structure of **2** was established. The 2D NMR correlations observed fully supported the locations of functional groups (Table 2).

**Figure 2:**
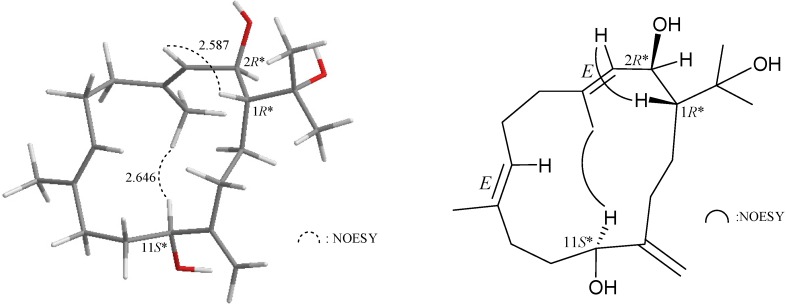
NOESY correlations of compound **2**.

In the NOESY experiment of **2** (Figure 2), H-1 correlated with H-3, but not with H-2, and large coupling constants were recorded between H-1/H-2 (*J* = 10.0 Hz) and H-2/H-3 (*J* = 9.2 Hz) and indicating that the dihedral angles between H-1/H-2 and H-2/H-3 are approximately 180° and the configurations of both chiral carbons C-1 and C-2 were assigned as R*. H-11 correlated with H_3_-18, but not with H_2_-20 confirming the S*-configuration of C-11 by modeling analysis. Furthermore, no correlation was found between H-3/H_3_-18 and H-7/H_3_-19, indicating that C-3/4 and C-7/8 carbon-carbon double bonds had an *E*-configuration.

**Table 2:** ^1^H (400 MHz, CDCl_3_) and ^13^C (100 MHz, CDCl_3_) NMR data, ^1^H–^1^H COSY and HMBC correlations for cembrane **2**.

Position	δ_H_ (*J* in Hz)	δ_C_, Multiple	^1^H–^1^H COSY	HMBC
1	1.54 m	51.9, CH	H-2, H_2_-14	
2	4.43 dd (10.0, 9.2)	71.7, CH	H-1, H-3	C-4
3	5.21 d (9.2)	128.3, CH	H-2, H_3_-18	C-5, -18
4		140.0, C		
5	2.26 m; 2.07 m	40.0, CH_2_	H_2_-6	C-3, -4, -7
6	2.26 m; 2.17 m	24.5, CH_2_	H_2_-5, H-7	C-4, -5, -7, -8
7	5.02 dd (6.8, 6.8)	124.5, CH	H_2_-6, H_3_-19	C-9, -19
8		134.6, C		
9	2.18 m; 2.03 m	33.9, CH_2_	H_2_-10	C-7, -8, -11, -19
10	1.75 m; 1.53 m	32.4, CH_2_	H_2_-9	C-11
11	4.08 dd (7.2, 4.0)	70.8, CH	H_2_-10, H-20a	C-12
12		152.0, C		
13	2.17–2.03 m	34.9, CH_2_	H_2_-14, H_2_-20	C-1, -11, -12, -14, -20
14	1.18 m; 1.07 m	27.4, CH_2_	H-1, H_2_-13	C-1, -2, -12, -13, -15
15		75.0, C		
16	1.29 s	30.3, CH_3_		C-1, -15, -17
17	1.31 s	24.2, CH_3_		C-1, -15, -16
18	1.73 s	15.7, CH_3_	H-3	C-3, -4, -5
19	1.58 s	16.2, CH_3_	H-7	C-7, -8, -9
20a/b	5.11 s; 4.85 s	109.6, CH_2_	H-11, H_2_-13	C-11, -12, -13

**Figure 3:**
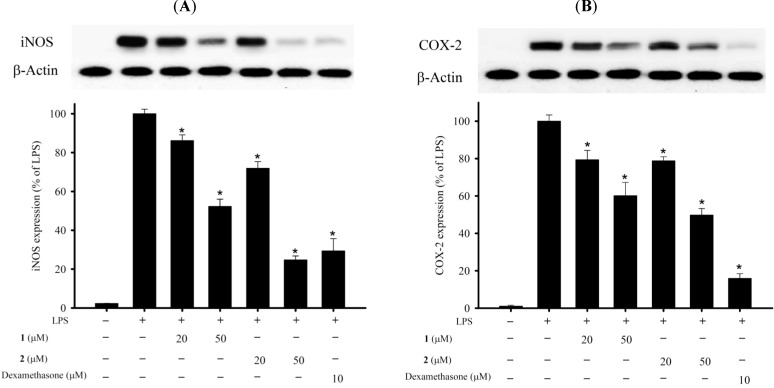
Effects of compounds **1** and **2** on pro-inflammatory inducible nitric oxide synthase (iNOS) and cyclooxygenase-2 (COX-2) protein expression in the lipopolysaccharide (LPS)-stimulated murine macrophage cell line, RAW264.7. (**A**) Relative density of iNOS immunoblot; (**B**) relative density of COX-2 immunoblot. The relative intensity of the LPS-stimulated group was taken to be 100%. Band intensities were quantified by densitometry and are indicated as the percent change relative to that of the LPS-stimulated group. Compounds **1**, **2** and dexamethasone (Dex) significantly inhibited LPS-induced iNOS protein expression in macrophages. The experiment was repeated three times (* *p* < 0.05, significantly different from the LPS-stimulated group).

In the *in vitro* anti-inflammatory activity test, the upregulation of the pro-inflammatory iNOS (inducible nitric oxide synthase) and COX-2 (cyclooxygenase-2) protein expression of LPS (lipopolysaccharide)-stimulated RAW264.7 macrophage cells was evaluated using immunoblot analysis. At a concentration of 50 μM, compounds **1** and **2** were found to significantly reduce the levels of iNOS and COX-2, respectively, relative to the control cells stimulated with LPS only (Figure 3). To evaluate the cytotoxic effects of compounds **1** and **2** on the viability of RAW264.7 macrophage cells, we used the alamar blue assay. Both compounds **1** and **2** (20 and 50 μM) did not significantly affect the viability of macrophage cells 16h after treatment. Thus, compounds **1** and **2** might be promising as anti-inflammatory agents, as they do not exhibit cytotoxicity to RAW264.7 macrophage cells.

**Table:** 

	**1**		**2**		Dexamethasone
μM	20	50	20	50	10
iNOS expression	86.14% ± 2.95%	52.26% ± 3.74%	71.88% ± 3.46%	24.74% ± 0.02%	29.34% ± 6.34%
COX-2 expression	79.38% ± 5.00%	60.17% ± 7.09%	78.79% ± 2.18%	49.79% ± 3.56%	15.97% ± 2.50%

^a^ Dexamethasone was used as a positive control.

Cytotoxicity of cembranes **1** and **2** toward MOLT-4 (human acute lymphoblastic leukemia), SUP-T1 (human T-cell lymphoblastic lymphoma), DLD-1 (human colorectal adenocarcinoma), LNCaP (human prostatic carcinoma) and MCF7 (human breast adenocarcinoma) tumor cells were studied, and the results are shown in Table 3. LNCaP cell line was more sensitive to the cytotoxic effects of **1**.

**Table 3:** Cytotoxic Data of Cembranes **1** and **2**.

Compounds	Cell Lines IC_50_ (μg/mL)				
	MOLT-4	SUP-T1	DLD-1	LNCap	MCF7
**1**	NA	NA	NA	9.80	NA
**2**	NA	NA	NA	NA	NA
Doxorubicin ^a^	0.001	0.19	0.08	2.68	1.39

^a^ Doxorubicin was used as a positive control and NA = not active at 20 μg/mL for 72 h.

## 3. Experimental Section

### 3.1. General Experimental Procedures

Optical rotation values were measured with a Jasco P-1010 digital polarimeter (Japan Spectroscopic Corporation, Tokyo, Japan). IR spectra were obtained on a Varian Digilab FTS 1000 FT-IR spectrophotometer (Varian Inc., Palo Alto, CA, USA); absorptions are reported in cm^−^^1^. NMR spectra were recorded on a Varian Mercury Plus 400 NMR spectrometer (Varian Inc., Palo Alto, CA, USA) using the residual solvent (CDCl_3_, δ_H_ 7.26 ppm for ^1^H NMR and δ_C_ 77.1 ppm for ^13^C NMR) as the internal standard. Coupling constants (*J*) are given in Hz. ESIMS and HRESIMS were recorded using a Bruker 7 Tesla solariX FTMS system (Bruker, Bremen, Germany). Column chromatography was performed on silica gel (230–400 mesh, Merck, Darmstadt, Germany). TLC was carried out on precoated Kieselgel 60 F_254_ (0.25 mm, Merck, Darmstadt, Germany); spots were visualized by spraying with 10% H_2_SO_4_ solution followed by heating. Normal phase HPLC (NP-HPLC) was performed using a system comprised of a Hitachi L-7110 pump (Hitachi Ltd., Tokyo, Japan), a Hitachi L-7455 photodiode array detector (Hitachi Ltd., Tokyo, Japan) and a Rheodyne 7725 injection port (Rheodyne LLC, Rohnert Park, CA, USA). A normal phase column (Supelco Ascentis^®^ Si Cat #: 581515-U, 25 cm × 21.2 mm, 5 μm, Sigma-Aldrich, St. Louis, MO, USA) was used for HPLC. The reversed phase HPLC (RP-HPLC) was performed using a system comprised of a Hitachi L-2130 pump (Hitachi Ltd., Tokyo, Japan), a Hitachi L-2455 photodiode array detector (Hitachi Ltd., Tokyo, Japan), a Rheodyne 7725 injection port (Rheodyne LLC, Rohnert Park, CA, USA). A reversed phase column (Supelco Ascentis^®^ Si Cat #: 581343-U, 25 cm × 10.0 mm, 5 μm, Sigma-Aldrich, St. Louis, MO, USA) was used for RP-HPLC.

### 3.2. Animal Material

Specimens of the octocoral *Nephthea columnaris* (Studer, 1895) were collected originally by hand using SCUBA equipment off the coast of the Southern Taiwan, and transplanted to five 0.6-ton cultivating tanks equipped with a flow-through sea water system in February 2012. The cultured octocorals for this research work were collected from the tanks in May 2013. Prof. Chang-Feng Dai, Institute of Oceanography, National Taiwan, identified the soft coral. Living reference specimens are being maintained in the authors’ marine organism cultivating tank and a voucher specimen (NMMBA-TWSC-12005) was deposited in the National Museum of Marine Biology and Aquarium, Taiwan.

### 3.3. Extraction and Isolation

Sliced bodies of *Nephthea columnaris* (wet weight 800.0 g, dry weight 76.6 g) were extracted with a mixture of MeOH and DCM (1:1) (1.6 L × 5). The extract was partitioned between EtOAc and H_2_O. The EtOAc layer (7.4 g) was separated on silica gel and eluted using *n*-hexane/EtOAc (stepwise, 100:1–pure EtOAc) to yield 17 fractions A–Q. Fraction J was chromatographed on NP-HPLC using a mixture of *n*-hexane and acetone (2:1) to afford 14 fractions J1–J14. Fraction J3 was separated by NP-HPLC using a mixture of *n*-hexane and acetone (2:1) as the mobile phase to yield 6 fractions J3A–J3F. Fraction J3B was purified by RP-HPLC using a mixture of acetonitrile and water (1:1) as the mobile phase to afford **1** (2.3 mg, *t*_R_ = 22 min). Fraction J5 was purified by RP-HPLC using a mixture of MeOH and water (3:2) as the mobile phase to afford **2** (2.0 mg, *t*_R_ = 22 min).

Columnariol A (**1**): Colorless oil; ${\left[\alpha \right]}_{\text{D}}^{23}$ + 90 (*c* 0.8, MeOH); IR (neat) ν_max_ 3419 cm^−1^; ^1^H (400 MHz, CDCl_3_) and ^13^C (100 MHz, CDCl_3_) NMR data, see Table 1; ESIMS: *m*/*z* 345 [M + Na]^+^; HRESIMS: *m*/*z* 345.23989 (calcd for C_20_H_34_O_3_ + Na, 345.24002).

Columnariol B (**2**): Colorless oil; ${\left[\alpha \right]}_{\text{D}}^{23}$ −53 (*c* 0.7, MeOH); IR (neat) ν_max_ 3355 cm^−1^; ^1^H (400 MHz, CDCl_3_) and ^13^C (100 MHz, CDCl_3_) NMR data, see Table 2; ESIMS: *m*/*z* 345 [M + Na]^+^; HRESIMS: *m*/*z* 345.23977 (calcd for C_20_H_34_O_3_ + Na, 345.24002).

### 3.4. In Vitro Anti-Inflammatory Assay

According to our previous and other studies for the *in vitro* anti-inflammatory activity assay, we used LPS induced RAW murine macrophage cell line which was purchased from American Type Culture Collection (ATCC, Manassas, VA, USA) [4,5,6,7]. The *in vitro* anti-inflammatory activity of Compounds **1** and **2** was measured by examining the inhibition of lipopolysaccharide (LPS)-induced upregulation of pro-inflammatory iNOS (inducible nitric oxide synthase) and COX-2 (cyclooxygenase-2) protein expression in macrophage cells using Western blotting analysis [7,8,9]. Briefly, inflammation in macrophages was induced by incubating them for 16 h in a medium containing only LPS (10 ng/mL) without compounds. For the anti-inflammatory activity assay, Compounds **1**, **2** and dexamethasone (10 μM) were added to the cells 10 min before the LPS challenge. The cells were then for western blot analysis. The immunoreactivity data were calculated with respect to the average optical density of the corresponding LPS-stimulated group. RAW264.7 macrophage cells viability was determined after treatment with alamar blue (Invitrogen, Carlsbad, CA, USA), the tetrazolium dye that is reduced by living cells to fluorescent products. This assay is similar in principle to the cell viability assay using 3-(4,5-dimethyldiazol-2-yl)-2,5-diphenyltetrazolium bromide and has been validated as an accurate measure of the survival of RAW264.7 macrophage cells [10,11]. For statistical analysis, the data were analyzed by a one-way analysis of variance (ANOVA), followed by the Student-Newman-Keuls *post hoc* test for multiple comparisons. A significant difference was defined as a *p*-value of <0.05.

### 3.5. MTT Antiproliferative Assay

Based on our previous and other studies from the cytotoxicity, there are different tumor cell lines (include MOLT-4, SUP-T1, DLD-1, LNCaP and MCF7 cells) which were obtained from ATCC [12,13,14,15,16]. Cells were maintained in RPMI 1640 medium supplemented with 10% fetal calf serum, 2 mM glutamine and antibiotics (100 units/mL penicillin and 100 μg/mL streptomycin) at 37 °C in a humidified atmosphere of 5% CO_2_. Cells were seeded at 4 × 10^4^ per well in 96-well culture plates before treatment with different concentrations of the tested compounds. In cell culture experiments, compounds **1** and **2** were dissolved in 100% dimethyl sulfoxide (DMSO) (clear). The final concentration of DMSO in the culture medium was 0.02% and the compounds were made concentrations of 1.25, 2.5, 5, 10 and 20 μg/μL prior to the experiments. After treatment for 72 h, the cytotoxicity of the tested compounds was determined using a MTT cell proliferation assay (thiazolyl blue tetrazolium bromide, Sigma-M2128). The MTT is reduced by the mitochondrial dehydrogenases of viable cells to a purple formazan product. The MTT-formazan product was dissolved in DMSO. Light absorbance values (OD = OD_570_ − OD_620_) were recorded at wavelengths of 570 and 620 nm using an ELISA reader (Anthos Labtec Instrument, Salzburg, Austria) to calculate the concentration that caused 50% inhibition (IC_50_), *i.e.*, the cell concentration at which the light absorbance value of the experiment group was half that of the control group. These results were expressed a percentage of the control ± SD established from *n* = 4 wells per one experiment from three separate experiments.

## 4. Conclusions

Octocorals have been well-recognized as an important source of potential bioactive marine natural products. However, because of the octocorals are claimed to be threatened species and most of the compounds from octocorals are difficult to obtain by chemical methods at this stage, bioactive substances from cultured marine invertebrates will play an important role in this field. Our studies on *Nephthea columnaris* for the extraction of additional natural substances, have led to the isolation of two new cembranes, colummnariols A (**1**) and B (**2**). Compounds **1** and **2** are potentially anti-inflammatory and may become lead compounds in future marine anti-inflammation drug development [17,18]. To the best of our knowledge, this is the first time to study the natural products from *N. columnaris*. These results suggest that continuing investigation of novel secondary metabolites together with the potentially useful bioactivities from this marine organism are worthwhile for future drug development. The octocoral *Nephthea columnaris* will be transplanted to culturing tanks located in the National Museum of Marine Biology and Aquarium, Taiwan, for extraction of additional natural products to establish a stable supply of bioactive material [19].
